# Comment on Mavroudis et al. Post-Traumatic Epilepsy After Mild and Moderate Traumatic Brain Injury: A Narrative Review and Development of a Clinical Decision Tool. *Reports* 2025, *8*, 193

**DOI:** 10.3390/reports8040233

**Published:** 2025-11-13

**Authors:** Roy G. Beran

**Affiliations:** 1 Global Health Neurology Lab, Sydney, NSW 2150, Australia; roy.beran@unsw.edu.au; 2 UNSW Medicine and Health, University of New South Wales (UNSW), South West Sydney Clinical Campuses, Sydney, NSW 2170, Australia; 3 School of Medicine, Western Sydney University, Sydney, NSW 2000, Australia; 4 Griffith Health, School of Medicine and Dentistry, Griffith University, Southport, QLD 4215, Australia; 5 Ingham Institute for Applied Medical Research, Clinical Sciences Stream, Liverpool, NSW 2170, Australia

The paper by Mavroudis et al. [1], as appears in this journal, takes a fresh look at post-traumatic epilepsy, as occurs following mild-to-moderate traumatic brain injury (TBI). It acknowledges that seizures may occur from months to years following TBI and hypothesises a method that may help delineate the associated risks and suggests how to determine the best algorithm for intervention.

Mild TBI is defined by a Glasgow Coma Scale (GCS) score of 13–15 post-TBI, and moderate TBI as having a GCS score of 9–12. Seizures, post-mild TBI, were said to have a 1–10% occurrence, and post-moderate TBI a 6–12% occurrence, showing some considerable overlap. Predictive factors include early seizure, cerebral contusions affecting frontal or temporal lobes, subdural haematomas, multiple contusions, and midline shift. Other factors include prolonged consciousness loss, male gender, psychiatric comorbidity, and electroencephalographic (EEG) abnormalities. Each of these features seems self-evident but it is timely to reiterate those factors which might predicate earlier intervention and more rigorous follow-up.

The importance of identifying these risk factors is to initiate a heightened level of awareness, especially in those who experience repeated TBI, even if it is mild. There have been a number of recent papers that examined TBI [2,3] with special reference to the legal and medicine ramifications thereof. Mavroudis et al. [1] emphasise the multifactorial pathogenesis for post-traumatic epilepsy and the potential use of artificial intelligence in monitoring affected individuals. This has great relevance in providing optimal supervision for those involved in contact sports and within the military, two areas where TBI is most prevalent. It cannot be overemphasised that even mild TBI, compounded by additional factors, such as imaging abnormalities or early (rather than immediate) seizures, may carry a risk of seizures up to 10%, not including the increased risk that may be associated with repeated mild TBI [4]. Tubi and colleagues [5] confirm that early onset seizures and temporal lobe trauma are powerful predictors of more sinister epilepsy [5], a finding supported by Mavroudis et al. [1]. Cerebral imaging is also an important factor in predicting post-traumatic seizures, especially those involving the temporal lobes [1,6].

The paper by Mavroudis et al. [1] provides a timely reminder that even mild TBI carries a significant risk of post-traumatic epilepsy, especially if accompanied by other contributing factors, and it provides food for thought to orchestrate early intervention where such risk of seizures becomes significant. Even mild TBI, in the presence of extenuating factors, should be treated with respect and possibly, prophylactic antiseizure medication. Consideration of these factors may also play a significant role in the later development of chronic traumatic encephalopathy and should possibly contribute to the earlier consideration of traumatic encephalopathy syndrome.
